# From unloading to trimming: studying bruising in individual slaughter cattle

**DOI:** 10.1093/tas/txaa165

**Published:** 2020-09-08

**Authors:** Helen C Kline, Zachary D Weller, Temple Grandin, Ryan J Algino, Lily N Edwards-Callaway

**Affiliations:** 1 Department of Animal Sciences, Colorado State University, Fort Collins, CO; 2 Department of Statistics, Colorado State University, Fort Collins, CO; 3 Animal Welfare, JBS Beef Division, Greeley, CO

**Keywords:** bruises, cull cows, traumatic event, trim loss

## Abstract

Livestock bruising is both an animal welfare concern and a detriment to the economic value of carcasses. Understanding the causes of bruising is challenging due to the numerous factors that have been shown to be related to bruise prevalence. While most cattle bruising studies collect and analyze data on truckload lots of cattle, this study followed a large number (*n* = 585) of individual animals from unloading through postmortem processing at five different slaughter plants. Both visual bruise presence and location was recorded postmortem prior to carcass trimming. By linking postmortem data to animal sex, breed, trailer compartment, and traumatic events at unloading, a rich analysis of a number of factors related to bruise prevalence was developed. Results showed varying levels of agreement with other published bruising studies, underscoring the complexity of assessing the factors that affect bruising. Bruising prevalence varied across different sex class types (*P* < 0.001); 36.5% of steers [95% confidence interval (CI): 31.7, 41.6; *n* = 378], 52.8% of cows (45.6, 60.0; 193), and 64.3% of bulls (no CI calculated due to sample size; 14) were bruised. There was a difference in bruise prevalence by trailer compartment (*P* = 0.035) in potbelly trailers, indicating that cattle transported in the top deck were less likely to be bruised (95% CI: 26.6, 40.4; *n* = 63) compared to cattle that were transported in the bottom deck (95% CI: 39.6, 54.2; *n* = 89). Results indicated that visual assessment of bruising underestimated carcass bruise trimming. While 42.6% of the carcasses were visibly bruised, 57.9% of carcasses were trimmed due to bruising, suggesting that visual assessment is not able to capture all of the carcass loss associated with bruising. Furthermore, bruises that appeared small visually were often indicators of larger, subsurface bruising, creating an “iceberg effect” of trim loss due to bruising.

## INTRODUCTION

Bruises not only impact overall carcass value but also raise concerns about cattle welfare and, therefore, bruising has received considerable interest within the cattle industry. The 2016 National Beef Quality Audit (NBQA) reported a bruise prevalence of 38.8%, 64.1%, and 42.9% for finished steer and heifer, cow, and bull carcasses, respectively (Eastwood et al., 2017; Harris et al., 2017). Similarly, in a large commercial study exploring risk factors associated with bruising in the United States, Lee et al. (2017) reported that 68.2% of cattle observed were bruised. Bruised areas on the carcass must be removed during postmortem processing resulting in decreased carcass value from reduced carcass yield and, depending on bruise location, potential devaluing of cuts. Industry reports have estimated that bruising costs the cattle industry millions of dollars in lost carcass value annually (Henderson, 2016). Although data from the past several NBQAs have indicated general reductions in bruise prevalence, there are still opportunities for improvement (Eastwood et al., 2017; Harris et al., 2017). Additionally, the NBQA reported that critical and extreme bruises made up 2.4% and 6.3% of bruises in finished steers and heifers and cull cows and bulls, respectively (Eastwood et al., 2017; Harris et al., 2017). Although those may be relatively low percentages, the severity of those bruises and impacts on animal welfare warrant further exploration.

The significance of the economic loss associated with bruising in addition to the animal welfare impacts of bruising has led to increased study of transport and preslaughter management to identify where bruising is occurring within the marketing process and reduce the overall prevalence of bruising in cattle. Identifying the causes of carcass bruising is challenging due to the numerous opportunities for bruising to occur within the supply chain. During marketing, cattle are exposed to many factors that have the potential to impact bruise prevalence at slaughter (Warriss, 1990). Rough handling, mixing with horned animals, transport through auction markets, transport distance, density during transport, and facilities in poor condition have all been shown to increase the risk of bruising in cattle (Shaw et al., 1976; Huertas et al., 2010, 2018; Strappini et al., 2010, 2012; Mendonça et al., 2016; Bethancourt-Garcia et al., 2019; Knock and Carroll, 2019; Lima et al., 2019). Furthermore, these factors can interact with one another and have a cumulative effect on bruising risk. The aforementioned factors, and the multiple time points in the marketing process during which cattle might be exposed to them, have made it difficult to pinpoint the crucial events within the supply chain that impact bruising the most. As a result, there is still a need to further understand the effects of these factors and identify the critical time points when bruising may be occurring. In addition to the challenge of separating the multitude of factors influencing bruise prevalence, studying bruising is also a challenge because information about bruising is not available until postmortem processing has occurred, i.e., connecting preslaughter factors with postmortem bruising requires a significant data-collection effort.

One approach used to identify the factors and time points associated with bruise prevalence is to study a couple of relevant factors and explore distinct phases of the process, working backward along the supply chain (i.e., beginning with slaughter and working backward, initially including unloading, next including transport and next including loading, etc). Lee et al. (2017) conducted a large commercial study to explore the relationship between traumatic events at unloading with the prevalence of bruising postmortem in finished cattle. Researchers reported 20.4% (SEM: + 1.1%) traumatic event prevalence and 68.2% (SEM: +1.2%) bruise prevalence but did not identify an association between traumatic events and carcass bruising. In the Lee et al. (2017) study researchers measured the prevalence of characteristics at the lot level (a lot being a specific group of cattle arriving at the facility together and remaining grouped together throughout the slaughtering process) but not at the individual animal level. Therefore, if an animal experienced a traumatic event there was no direct reporting of that individual’s bruise score, just the score for the overall lot. Collecting data on lots of cattle is the method commonly utilized in studies like this because following individual animals through the entire slaughtering process is challenging (Lee et al., 2017; Mendonça et al., 2018; Bethancourt-Garcia et al., 2019). Although Lee et al. (2017) did not report any correlation between a traumatic event and bruise prevalence, they did report a relationship between trailer type, breed type, and traumatic events, identifying this as an area to explore in future studies.

There has been considerable industry discussion and concern about how animal characteristics may impact bruise prevalence. Multiple studies have reported sex class as a factor associated with increased bruising, but many studies relate this finding to differences in behavior between sex classes rather than physical characteristics or breed type (Mendonça et al., 2018; Knock and Carroll, 2019; Lima et al., 2019). The two primary factors associated with animal type that have been identified as potentially influencing the risk of increased bruising during the marketing process are the condition of culled cattle and the height of Holstein animals. The 2016 NBQA reported that cull cows have a greater percentage of bruising as compared to finished cattle. Furthermore, cull cows had greater percentages of critical (visually estimated as 5.0–18.14 kg in size) and extreme (visually estimated entire primal trimmed) bruises (Harris et al., 2017). Additionally, Lee et al. (2017) found that Holsteins had higher bruise prevalence than non-Holstein finished cattle. That study also found that there was an interaction of trailer type with animal type. Holsteins had a greater prevalence of bruising in trailers that had lower clearance into the bottom deck of the trailer, likely due to the Holsteins’ greater height relative to non-Holstein cattle. Further understanding the impacts of breed type and sex class on bruise prevalence would help provide strategies for reducing postmortem bruising.

Past research has focused primarily on the preslaughter experience of a lot or load of cattle rather than looking at individual animal data. Although this information is valuable for benchmarking in large groups of cattle, this type of study does not provide the level of detail necessary to link specific events, such as jamming during unloading, to specific incidents of bruising. Additionally, the available literature on bruising only reports bruise prevalence and does not report subsequent carcass trimming. The economic loss from a bruise is related to the trim that is removed from the carcass. The relationship between the frequency of bruising on carcasses determined by visual assessment and subsequent carcass trimming has not been studied.

The current study is unique in that it followed individually identified animals from unloading through postmortem processing at multiple slaughter facilities. This study assessed the relationship between preslaughter factors, specifically unloading, trailer compartment during transport, and origin, and characteristics of cattle, specifically breed type and sex class, with subsequent bruise presence and occurrence of carcass trimming. This study provides an assessment of the relationship between cattle type, sex class, origin location, and trailer compartment and bruise prevalence.

## MATERIAL AND METHODS

### Ethical Statement

All animal measurements were noninvasive observations of commercial industry practices. An exemption petition was filed and granted by the Colorado State University Animal Care and Use Committee for this study.

### Description of Slaughter Facilities and Study Animals

This study was conducted at five commercial cattle slaughter facilities across the United States that processed both cull cows and bulls and finished steers. Data were collected during 1 wk at each facility from October 2017 to March 2018. The slaughter facilities were single-production shift plants, operating one 9-h shift and slaughtering approximately 1,100 to 1,950 cattle per day. The chain speed ranged from 140 to 280 animals per hour across the five facilities. A total of 585 individually identified cattle were included in this study. Transport distances for cattle included in the study ranged from 96.6 to 1,367.9 km. Driver experience ranged from less than 1 mo to 45 yr.

### Animal Identification

Ten trucks arriving at the facility each night from 1800 to 400 hours were selected for study inclusion. Trucks chosen for study inclusion followed standard plant unloading procedures. The animals on these trucks would be the first slaughtered the following day. From each sampled truck, five animals were individually marked during unloading for postmortem data collection. Animals were selected to get a representative sample from each trailer compartment. Additionally, marking the lead animal to exit the trailer from each compartment was avoided to prevent balking and delays. Otherwise, animal selection was random. Animals arrived in both “potbelly” double-deck trailers and straight-deck trailers. Potbelly trailers have five compartments, two large top and bottom decks and three smaller compartments in the nose and tail. The straight-deck trailers were single deck and did not require cattle to climb up or down a ramp to load. Three methods were utilized to mark animals, depending on facility layout: food-grade dye (Grade and Yield Ink, Packers Chemical, Kieler, WI) applied on the dorsal topline with a sprayer (RL Flo-Master 1-Gallon Sprayers, Lowell, MI), a sticky patch (Estrotect Heat Detector, Rockway Inc., Spring Valley, WI) applied with adhesive to the back of the animal (Kamar Adhesive, Kamar Products Inc., Zionsville, IN), or livestock marker paint sticks (All Weather Paint Stik, La-Co Industries, Elk Grove Village, IL) attached to a sorting pole (Sorting Poles, U.S. Whip, Miami, OK). Multiple colors of each medium were used to designate the compartment and specific trailer load. One researcher was responsible for marking the animals as they exited the trailer. The following data were recorded for the individual marked animals: trailer compartment of origin, number of nonambulatory animals (downers), number of animals dead on arrival, animal sex class (cull cow, finished steer, or cull bull), total number of animals in the trailer, and breed type (classified as either beef breed or dairy breed), distance traveled, and driver experience (years). Additional identifiers were recorded for the individually marked animals to assist with identification postmortem: back tag number, ear tag number, and hide color. From the 10 trucks sampled, approximately 50 individual animals were marked each night over the 3 d of sampling at each facility, totaling a target of 150 individually identified cattle per facility.

### Unloading Procedure and Assessment

Four GoPro Hero 5 Black Series cameras (GoPro, San Mateo, CA) fitted with 64 gigabyte 4K SD cards (Western Digital Technologies, Inc., Milpitas, CA) were used each night to record the unloading process for each of the sampled trailers. Two cameras were placed on each unloading dock (the number of unloading docks was plant dependent), with GoPro Jaw Clamps (GoPro, San Mateo, CA) to capture two different views: 1) the exit and inside of the trailer and 2) the observer marking the cattle. The camera locations were selected to capture the entire exit of the trailer with no obstruction of animals exiting the trailer. The exact camera location varied by the facility and was determined during project preparation.

After completing data collection at each facility, the video footage of unloading was reviewed. The video reviewer recorded whether or not a traumatic event occurred at unloading and the location on the body that the trauma occurred. A traumatic event was counted when an animal hit any part of the trailer during the unloading process (Lee et al., 2017).

After the cattle were unloaded, they were moved to a designated lairage pen by plant employees where they were held until plant processing began in the early morning. Cattle remained in these pens until that group (i.e., lot) of cattle was scheduled for slaughter the following morning. A USDA veterinarian conducted antemortem inspection of the cattle before the animals were moved through the facility to the restrainer where they were stunned. All cattle were stunned using a pneumatic captive bolt gun (Jarvis Products Corporation, Middletown, CT). One facility used a stun box, but all other facilities utilized a center track restrainer.

### Visual Bruise Assessment and Trim Collection

Researchers observed and tracked animal carcasses during multiple stages of the slaughter process. These carcasses included both those from animals individually marked and from other animals that were present on the selected trucks. To maintain the identity of the animals that had been identified and individually marked at unloading and track their carcasses, researchers added uniquely identifying tags at multiple processing stages.

Observers recorded carcass bruising information after the hide was removed, but before the carcasses were split. At this point, observers recorded bruise presence (yes/no) and, if bruises were present, the bruise location. A modified version of the Strappini et al. (2012) diagram was used to score bruise location. The original Strappini et al. (2012) diagram had a single long section along the backbone, and our modified diagram divided the back into three sections for further delineation of bruise location. Bruise location was also recorded. The same observer visually scored bruises at every facility in this study. The observer scored not only the individually identified carcasses but also other carcasses that were processed during the data-collection time frame. Only bruise presence and location were recorded for this larger sample of carcasses (*n* = 8,959).

After visual bruise assessment, all bruise trimming was removed by plant employees following standard facility procedures. Researchers were positioned at the final trim rail, where carcasses were assessed for contamination or damage. Bruise trimmings were collected from each individually identified carcass. Bruise trim was defined as a damaged area that had a focal point of discoloration caused by blood collecting under the surface of the epidermis, which could be seen with the naked eye (Marshall, 1977; Capper, 2001; Langlois, 2007; Pilling et al., 2010). If a carcass was not trimmed, for example, had zero material removed, that was also recorded. As trim was removed from the carcass, a researcher placed the trim from each identified carcass into a labeled plastic bag. The bag was sealed and placed in a container for subsequent weighing at the end of the collection period (data not included in this paper).

### Statistical Analysis

Data were analyzed using the software R (R Core Team, 2019). Analyses included summary statistics, calculation of confidence intervals (CIs), and chi-square tests and logistic regression to draw inferences from the sample data. Data were analyzed to evaluate differences in bruising prevalence rates as a function of trailer compartment, breed type, origin, and sex class. Analyses were also conducted to assess the relationship between driver experience and haul distance and bruising prevalence. Due to challenges with keeping track of individual carcasses throughout the stages of processing and subsequent data-quality control checks, 585 of the targeted 750 individually identified animals were included in final analyses of bruise risk factors and trimming. The larger sample of 8,959 carcasses, many of which were not individually identified before unloading, were used to assess visual bruise prevalence and bruise locations.

## RESULTS AND DISCUSSION

Previously published studies in the United States and Canada have reported bruise prevalence ranging from 40% and 68%, with percentages traditionally reported by animal sex class, that is, cull cows and bulls versus finished steers and heifers (Van Donkersgoed et al., 2001; Eastwood et al., 2017; Harris et al., 2017; Lee et al., 2017). Studies outside of North America have reported larger variation in bruise prevalence among studies, ranging from 8% to 99% (Jarvis et al., 1995; Marshall, 1977; Gallo et al., 1999; Costa et al., 2006; Andrade et al., 2008; Strappini et al., 2010; Romero et al., 2013; Huertas et al., 2015; Bethancourt-Garcia et al., 2019). The considerable variations in sample size, size of slaughter plant, bruise severity, scoring system, animal type, and management procedures of studies likely contributes to the substantial spread in bruise prevalence across studies. In this study, the overall visible bruising prevalence of the larger sample size (*n* = 8,959) was 28.1% and the bruise prevalence for the individually identified cattle sample (*n* = 585) was 42.6%. Detailed information on the characteristics (e.g., breed type and sex class) of the larger population was not collected and, thus, explaining this difference in bruise prevalence between the two samples is challenging. The authors hypothesize that the difference could potentially be due to the different population demographics between samples, specifically differences in factors that influence bruising, such as breed type and sex class.

**Figure 1.**
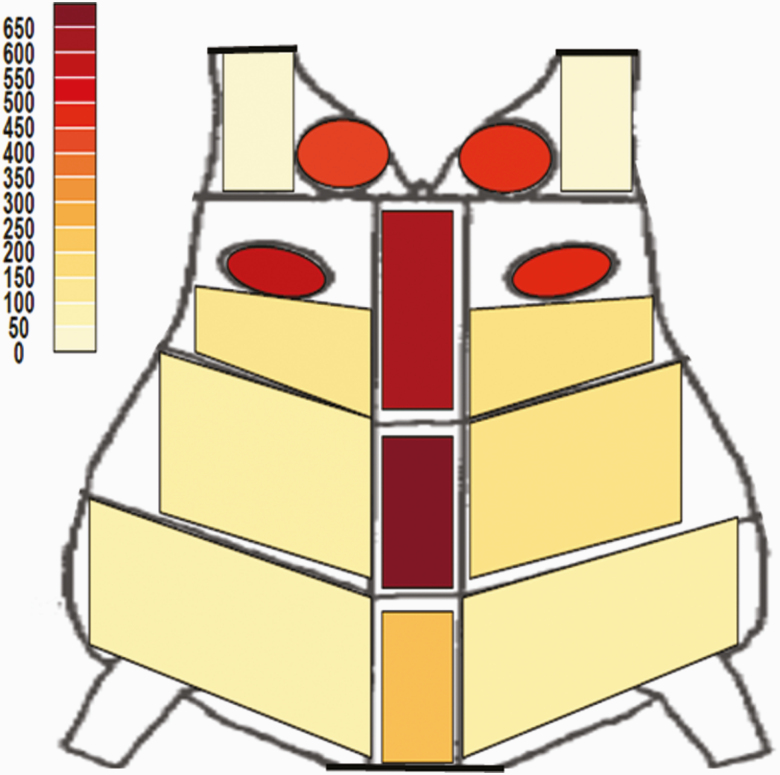
Bruise counts by carcass location (*n* = 8,959). The darker the color in the figure, the greater the number of total bruises found in the region. The image is adapted from Strappini et al. (2012).

In the current study, the greatest frequency of bruising was found along the dorsal midline and in the rump region (Figure 1), as similarly reported in other studies (Jarvis et al., 1995; Romero et al., 2013; Huertas et al., 2015; Eastwood et al., 2017; Harris et al., 2017; Lee et al., 2017). There are several possible explanations for bruises being most concentrated in these regions. Increasing cattle size and frame, coupled with trailer design, and improper use of vertically closing gates have been suggested as potential reasons for greater bruising in these specific carcass regions (Grandin, 1980; Blackshaw et al., 1987; Strappini et al., 2009; Gray et al., 2012; Huertas et al., 2015; Lee et al., 2017).

There have been numerous studies exploring potential risk factors impacting bruise prevalence and many of the studies have reported differing results. Table 1 provides a summary of sample characteristics, including breed type, sex class, traumatic events, cattle location of origin, and trailer type for the individually marked animals. In the current study, there was no difference in bruise prevalence by the presence of traumatic events experienced during unloading (*P* = 0.168). Of the cattle experiencing a traumatic event, 59.1% were bruised (95% CI: 36.7, 78.5; *n* = 22) and, of those not experiencing a traumatic event, 41.9% were bruised (37.8, 46.1; *n* = 563). This particular study did not attempt to relate carcass bruise location to the region of the animal’s body impacted by a traumatic event antemortem, but this type of assessment would be beneficial for establishing an improved understanding of the relationship between handling and bruising.

**Table 1.** Summary of experimental animal characteristics (*n* = 585)

Characteristic	*n* (% of sample)
Breed type	
Beef breed	140 (23.9)
Dairy breed	445 (76.1)
Sex class	
Bull	14 (2.4)
Cow	193 (33.0)
Steer/heifer	378 (64.6)
Traumatic events	
No traumatic event	563 (96.2)
Traumatic event	22 (3.8)
Origin	
Auction market	419 (71.6)
Other location	165 (28.2)
Not recorded	1 (0.2)
Trailer type	
Potbelly trailers	132^*a*^ (95.7)
Straight trailers	6^*a*^ (4.3)

^*a*^The *n* for trailer type represents unique trailer rather than individual animal.

There was a difference in bruise prevalence by breed type (*P* = 0.011); 52.1% of beef type cattle were bruised (95% CI: 43.6, 60.6; *n* = 140) and 39.6% of dairy-type cattle were bruised (35.0, 44.3; 445; Table 2). Beef type cattle included bulls, cows, and steers, and dairy-type cattle included cows and steers. Lee et al. (2017) identified a relationship between breed type and bruise prevalence, reporting a greater frequency of bruising in Holstein as compared with non-Holstein cattle. Their study included steers and heifers, while this study also had culled animals within each breed type. Other studies have explored breed as a factor influencing bruising but are not easily comparable to this study as the breed types were different (e.g., Zebu and Simmental; Wythes et al., 1985; Hoffman and Lühl, 2012). Although there was a difference in bruise prevalence between beef and dairy breed types, the magnitude of this difference was not well constrained (12% difference, 95% CI: 2.7%–22.5%). Breed type is often related to physical characteristics (e.g., height, body condition, and temperament) and, therefore, future studies should focus on the qualities of breeds that may be associated with bruising.

**Table 2.** Bruise prevalence by identified potential risk factor (*n* = 585)

Factor	Total *n*	Cattle bruised within a subfactor *n* (%)	95% CI
Breed type			
Beef	140	73 (52.1)	43.6, 60.6
Dairy	445	176 (39.6)	35.0, 44.3
Sex class			
Bull	14	9 (64.3)	— ^*a*^
Cow	193	102 (52.8)	45.6, 60.0
Steer	378	138 (36.5)	31.7, 41.6
Traumatic events			
No traumatic event	563	236 (41.9)	37.8, 46.1
Traumatic event	22	13 (59.1)	36.7, 78.5
Origin			
Auction market	419	177 (42.2)	37.5, 47.1
Other location	165	71 (43.0)	35.4, 51.0
Not recorded	1	1 (100.0)	—
Trailer compartment^*b*^			
Belly	190	89 (46.8)	39.6, 54.2
Doghouse	9	5 (55.6)	— ^*a*^
Tail	102	49 (48.0)	38.1, 58.1
Nose	76	34 (44.7)	33.5, 56.5
Top deck	190	63 (33.2)	26.6, 40.4

^*a*^CI was not calculated due to a small sample size.

^*b*^The total *n* shown for trailer compartment is 567 animals. Animals arriving in straight trailers (*n* = 18) were not included due to a small sample size for that trailer type.

Previous research has reported variable results in regard to the impact of sex class on bruising prevalence (Weeks et al., 2002; Strappini et al., 2009; Romero et al., 2013; Bethancourt-Garcia et al., 2019; Mendonça et al., 2019). In this study, bruising prevalence varied across different sex class types (*P* < 0.001); within the study sample, 36.5% of steers (95% CI: 31.7, 41.6; *n* = 378), 52.8% of cows (45.6, 60.0; 193), and 64.3% of bulls (no CI calculated due to sample size; 14) were bruised (Table 2). Romero et al. (2013) found that males (mostly bulls) have the greatest risk of bruising compared to females, whereas Hoffman and Lühl (2012) demonstrated the opposite finding. It is essential to note that all of these studies vary greatly in animal type, both sex class, breed type, and research objectives. The variation in the literature underlines the complexity of bruising. No one factor alone impacts bruising and, therefore, it is difficult to assess how a single factor affects bruising and a multifactor approach is recommended. Particularly, in the United States, there is considerable industry discussion about differences in bruising between cull cows and finished steers and heifers. The NBQAs have consistently identified cull cows as not only having a higher bruise prevalence as compared with finished cattle but also demonstrating a greater percentage of critical and extreme bruising (Eastwood et al., 2017; Harris et al., 2017). As it is continually reported that culled animal populations have greater postmortem bruising, efforts on bruise reduction should include targeted focus for this population. Many of these culled animals are not shipped directly to slaughter, that is, they may be sold through an auction market prior to shipment to the slaughter plant, unlike finished animals that generally are directly shipped to slaughter plants (USDA, 2018). Despite this difference in transport to the plant between sex classes, place of origin (auction market or other) was not identified as a risk factor for bruising in the current study (*P* = 0.936). Within the study sample, 48.5% of cattle derived from auction barns were bruised (95% CI: 37.5, 47.1; *n* = 419) and 36.9% of cattle not derived from auction barn origin were bruised (35.4, 51.0; 165). Other studies assessing origin (usually comparing farm to auction market) have reported variable results, some identifying origin as a risk factor for bruising (Jarvis et al., 1995; Romero et al., 2013) and others showing no impact (Weeks et al., 2002; Strappini et al., 2009).

Another considerable difference between culled and finished cattle populations is the condition of the animal. Benchmarking studies have quantified the condition of cattle arriving at slaughter plants and identified several physical defects in cull cow populations, including, but not limited to, lameness, emaciation, poor udder condition, and prolapse (Harris et al., 2017; Vogel et al., 2018). Some of these characteristics may make these animals more prone to bruising during handling and transport both from physical attributes and the ability to move through the handling systems. Although data were collected on some physical characteristics like body condition score (BCS), there were very few observations for several BCS categories/breed type/sex combinations, which made it difficult to draw conclusions about how BCS impacted bruising. Future studies should be conducted to understand the complex relationships between animal characteristics, such as BCS, lameness, hock lesions, and udder score, to characterize how physical condition impacts bruising.

Although trailer compartment density, trailer type, and trailer condition have been studied as potential risk factors associated with carcass bruising (Eldridge et al., 1988; Tarrant et al., 1988, 1992; Huertas et al., 2010; Romero et al., 2013; Lee et al., 2017; Mendonça et al., 2018, 2019), these studies did not explore location within the trailer (trailer compartment) as a risk factor. In this study, the cattle transported in the bottom deck had greater bruising as compared to those on the top deck (*P* = 0.035; 46.8% and 33.2%, respectively) in potbelly trailers (Table 2). In personal communication with cattle haulers, some have shared that, during loading, cattle going down into the bottom deck of the trailer often bang their backs on the overhanging framework. Additionally, Lee et al. (2017) reported an interaction of trailer design and cattle type on traumatic event prevalence. They found a greater frequency of traumatic events observed in Holstein cattle hauled in a trailer that had a lower clearance into the bottom deck as compared with a trailer that had a greater clearance into the bottom deck.

In this study, there was no clear relationship between driver experience or haul distance and bruising prevalence. Logistic regression models were used to assess evidence for an increase in the likelihood of bruising with increases in haul distance and decreases in driver experience. For both haul distance (*P* = 0.34) and driver experience (*P* = 0.22), there was not enough evidence to suggest a relationship between these factors and bruise incidence.

One important finding in this study was that visual assessment of bruising was not a good indicator of whether or not a carcass was trimmed due to bruising. Bruise trim loss occurred on 57.9% of the sampled carcasses (Table 3). Of the sampled carcasses that were scored as visibly bruised, 77.9% were trimmed. However, 41.7% of carcasses that did not have visible bruising on the surface were trimmed. These findings suggest that visual assessment alone is potentially underestimating the actual lost carcass value associated with bruising. Additionally, from on-site observations, it was noticed that there were several instances in which slaughter plant employees were trimming large, deep bruises off the carcass that only appeared as small discolorations on the carcass surface. This observation suggests that the size of the bruise on the surface does not always accurately represent the actual size of the bruise. The authors refer to this phenomenon as the “iceberg effect” because the visual carcass bruising seen on the surface does not always reveal the magnitude of bruising that is beneath. The use of current visual bruise scoring systems could be underestimating bruise prevalence in cattle as the surface bruising may not always accurately represent the actual volume of bruise loss.

**Table 3.** Percentage of “bruised” and “not bruised” carcasses that were subsequently trimmed for bruising (*n* = 585)

Category	% of total carcasses (*n*)	% Trimmed within category (*n*)
Visually bruised	42.6% (249)	77.9% (*n* = 194)
Not visually bruised	57.4% (336)	43.2% (*n* = 145)
Total	100% (585)	57.9% (*n* = 339)

As demonstrated, bruising in cattle is impacted by multiple interacting factors associated with animal characteristics, such as breed type and sex class and preslaughter management practices, including animal handling and features of transport. Understanding when and how bruising occurs is a critical component to making reductions in bruise prevalence in the cattle industry. Future large-scale studies should include additional handling events into bruise prevalence risk factor analysis. For example, scoring traumatic event prevalence at both loading and unloading should be conducted to further elucidate the role of specific animal handling events in carcass bruising. Although researchers have attempted to pair live animal handling events with bruise location and severity information, this area of work should be expanded to include different methodologies, which would provide a more refined attribution of bruising to events in order to disentangle the complexity of factors affecting bruising. To expand upon some of the findings associated with trailer type and compartment, future studies should focus on events that occur within the trailer (e.g., bumping trailer structure during transport, falling, and movement into the bottom deck) to identify additional ways to mitigate potential bruising at this point in the process. Identifying critical control points for bruising in the livestock supply chain is an ongoing process as there are many contributing and interacting components. By examining each step in the transport and preslaughter management process, risk factors associated with increased bruising can be identified, which will help inform the development of bruise reduction techniques and programs.
